# Impact of a new palliative care consultation team on opioid prescription in a University Hospital

**DOI:** 10.1186/1472-684X-8-2

**Published:** 2009-01-16

**Authors:** Carlos Centeno, María Angustias Portela, Antonio Noguera, Antonio Idoate, Álvaro Sanz Rubiales

**Affiliations:** 1 Unidad de Medicina Paliativa y Control de Síntomas, Clínica Universidad de Navarra, Pamplona, Spain; 2 Unidad de Medicina Paliativa, Hospital Laguna, Madrid, Spain; 3 Departamento de Farmacia, Clínica Universidad de Navarra, Pamplona, Spain; 4 Unidad de Oncología, Hospital Universitario del Río Hortega, Valladolid, Spain

## Abstract

**Background:**

There are no validated measuring tools to gauge the effectiveness of a Hospital Palliative Care Consultation Team (PCCT). One way would be to consider its effect on the consumption of opioids expressed in total amounts and different formulations administered. We perform this study to evaluate the impact of a hospital PCCT on the trends of opioid prescription in a University Hospital.

**Methods:**

A seven year retrospective study on opioid prescription was carried out in the Clínica Universidad de Navarra. The period includes three years before and three years after the PCCT was implemented. Prescription was analysed calculating yearly the Defined Daily Dose (DDD) adjusted to 1000 hospital stays (DDD/1000HS). Indicators considered were the proportion of patients treated using opioids compared to the total estimated in need of treatment (rate of effectiveness) and the proportion of patients potentially requiring opioids but not treated who were incorporated into the treatment group (rate of improvement).

**Results:**

From 2001 to 2007, total opioid prescription was low in non-oncology Departments (range: 69–110 DDD/1000HS) while parenteral morphine and fentanyl did not register any changes. In the same period of time, total opioid prescription increased in the Oncology Department from 240 to 558 DDD/1000HS. The rate of effectiveness in the three years prior to the implantation of the consultation team was 64% and in the three following years rose to 87%. The rate of improvement prior to the palliative care consultation team was 43% and in the three following years was 64%. A change in opioid prescription was registered after the implementation of the PCCT resulting in an increase in the prescription of parenteral morphine and methadone and a decrease in transdermal fentanyl.

**Conclusion:**

Implementation of a PCCT in a University Hospital is associated with a higher and more adequate use of opioids.

## Background

Pain relief is one of the key issues for advanced-stage cancer patients and their carers. The World Health Organization (WHO) analgesic ladder recommended the use of weak and strong opioid to treat pain [1]. Data from the early 1990s described that 40% cancer patients do not receive sufficient pain relief [2,3]. However, in the last years there has been a steady increase in the prescription of opioids for the treatment of pain, especially in developed countries, including Spain [4].

There is evidence of the efficiency of interdisciplinary palliative care teams, based in hospitals and cancer centres [5]. A particular kind of these teams are the Palliative Care Consultation Team (PCCT) appeared in the 1970s. A PCCT, usually does not have patient beds under its responsibility but rather it acts in an advisory role to other healthcare professionals and works with the aim of bringing palliative care principles into acute care hospitals [6-8]. The aim of improving palliative care in all hospital patients is achieved by providing direct care by the team and by offering advice to attending doctors [9], as well as by trying to influence the work of other care professionals and staff members [10,11]. The goal is that patients in need of palliative care admitted to any department will receive appropriate care even without the direct intervention of a specialised palliative care team [12,13].

It is estimated that in the USA, 50% of hospitals have a palliative care team, or plan to set one up soon [14]. In Europe, the countries with the most developed Consultation teams are the United Kingdom and France where over 300 services of this type have been identified, corresponding to 35% and 65% of all palliative care units respectively [15]. Data exists which suggest that patients cared for by consultation teams have better assessment of problems and achieve better symptom management [16-18]. Nevertheless, the evaluation of the impact of these consultation teams has been generally limited only to those patients treated directly by the team, without taking into account the whole institution where their influence can be measured on a larger scale.

Pain is one of the most common symptoms occurring in cancer and for many years, opioid prescription has been used as an indicator of appropriate care [19]. In the same way, the impact of the consultation team may be measured in the changes in opioid prescription within the setting where they operate. It is reasonable to expect that their work may result in an increase in the total amount of opioids prescribed in the centre as a whole or at least in those departments targeted by the consultation team. In France, for example, part of the assessment of the hospital consultation teams includes analysing the total amount of opioids prescribed in the hospital where they operate.

In November 2004, a palliative PCCT started its work at the Clínica Universidad de Navarra, a 300 bed teaching hospital. Initially the team's activity was centred on cancer patients. The PCCT is integrated in the Oncology Department and consisted of two doctors, two nurses, a psychologist and a researcher; it provide support to the whole Oncology Department, composed of 21 oncologists. The PCCT dealt mainly with hospital inpatients, although over time, an increasing number of outpatients were also seen. During the course of its third year, it treated 20% of the 1842 inpatients at the Oncology Department that induced 10896 hospital stays.

There is no known level of opioid prescription which may be considered the ideal for a teaching hospital. In any case, a maximum can be estimated in function of the number of inpatients with severe pain. It can also be assumed that the consultation team has some influence on the type of opioids consumed in the hospital, in that the chosen medications and formulations will be expected to be those considered most effective and safe to meet the patients' needs.

This study was designed to measure the influence of a PCCT expressed as the impact on the trends of opioid prescription in a University Hospital. To do that, we analyse the patterns in opioid prescription for patients admitted to the Clínica Universidad de Navarra and design indicators of efficiency and effectiveness in pain treatment in cancer patients.

## Methods

A study was performed analyzing the amount and type of opioids prescribed to inpatients at the Oncology Department and other Departments at the Clínica Universidad de Navarra, between October 1^st ^2001 and September 30^th ^2007, covering the three years prior to and the three years following the setting up of the consultation palliative care team. Inpatients in departments different from Oncology were assigned to a control group. Opioids included in the study were those which can be administered at regular intervals (oral and parenteral morphine, oral and parenteral methadone, oxycodone and transdermal fentanyl). Opioids excluded were transmucous oral fentanyl, (use limited to breakthrough pain), intravenous fentanyl and remifentanil (used limited to diagnostic tests) and transdermal buprenorphine (not yet listed in the Hospital's Pharmacotherapy Guide).

The Pharmacy Department supplied the data on the quantity and formulations of opioids prescribed and dispensed to inpatients of the Oncology Department. Using this data, the Defined Daily Dose (DDD) for each opioid and for each administration route [20] was calculated. The DDD represents the average daily maintenance dose of a medication when it used routinely for its main indication and it is expressed as the amount of active ingredient in milligrams, according to its therapeutic equivalence and route of administration (Table 1) [21]. In the calculations for the DDD of transdermal fentanyl consideration was made only of the dose absorbed by the patient in three days and not the dosage in each patch.

**Table 1:** Equivalence in the Defined Daily Dose (DDD) for different opioids (see reference 20).

Opioid	DDD (mg)
Parenteral morphine	30
Oral morphine	100
Parenteral methadone	25
Oral methadone	25
Oral oxycodone	75
Transdermal fentanyl	1.2

The total number of DDD was adjusted to the annual length of stay in the Oncology Department in thousands. The following formula was applied: DDD per 1000 hospital stays divided by the total annual length of stay per year (DDD/1000HS = total DDD × 1000 × annual length of stay^-1^) per year.

In order to obtain indicators of efficiency and improvement in pain treatment an estimate on the number of patients admitted with cancer pain was calculated using the rationale that approximately 80% of patients with advanced stages suffer pain [22,23]. Since the Oncology Department already treats patients with diverse stages of the illness, it is reasonable to expect that the percentage of patients suffering from pain might be somewhere between 50% and 80%. For the purpose of this study, the higher figure was chosen. Of these cancer patients suffering pain that requires opioid treatment has been estimated to be around 80% [24,25]. This means that approximately 64% of all patients at the Oncology Department should be ideally treated with opioids.

Following this estimate, as DDD represents the average daily maintenance dose, as long as all those in need do indeed receive opioid treatment the ideal DDD per thousand cancer patients should move around 640. If we take this reference as valid, the efficiency of pain treatment for a cohort of cancer patients can be estimated by calculating the proportion of patients receiving opioid treatment in relation to the total calculated number in need. As a lineal relation can be estimated between total DDD and the number of patients receiving opioids, data of DDD prescribed to Oncology patients are an indicator of pain treatment efficiency. It is expressed in thousands of hospital stays per year and shown in relation to the ideal maximum estimated annual use and can be established as DDD/1000HS × 640^-1 ^(and multiplied × 100 to present the proportion as percentage).

Based on the estimated figure that 64% of cancer inpatients require opioids, it is possible to calculate the percentage of patients who should be receiving this kind of treatment and those who are not as well as estimate how this difference changes every year. This indicator of improvement in pain treatment is estimated as (Final DDD/1000HS - Initial DDD/1000HS) × (640 - Initial DDD/1000HS)^-1 ^(and multiplied × 100 to present the proportion as percentage).

## Results

Table 2 shows the DDD/1000HS for each opioid dispensed by the Pharmacy Department to the Oncology Department during the 2001–2007 period. As the use of meperidine in Oncology was minimal (14–43 units per year), it was excluded from this analysis. During the three years prior to initiation of the PCCT, the total prescription of opioids rose sharply from 240 to 411 DDD/1000HS. After three years it rose to 558 DDD-1000HS. Opioid consumption between 2001 and 2007 in the other Departments was much lower than the one observed in Oncology and demonstrated a steady trend (between 56 and 94 DDD/1000HS) (Table 3).

**Table 2:** Prescription of opioids in hospitalized patients in the Oncology Department in Daily Defines Doses per 1000 hospital stays (DDD/1000HS) per year.

Opioid	2001	2002	2003	2004	2005	2006	2007
Parenteral morphine	70	79	114	120	127	242	289
Oral morphine	52	43	48	56	55	70	65
Oxycodone	0	0	0	0	5	28	29
Parenteral methadone	8	4	5	6	3	0	4
Oral methadone	9	2	2	7	22	30	68
Transdermal fentanyl	101	160	157	222	184	158	103

Total DDD/1000HS	240	288	326	411	396	528	558

**Table 3:** Prescription of opioids in hospitalized patients in Departments others than Oncology in Daily Defined Doses per 1000 hospital stays (DDD/1000HS) per year.

Opioid	2001	2002	2003	2004	2005	2006	2007
Parenteral morphine	43	40	40	44	47	64	61
Oral morphine	3	1	2	2	2	3	2
Oxycodone	0	0	0	0	1	3	2
Parenteral methadone	4	1	1	0	0	1	0
Oral methadone	1	1	0	1	1	2	3
Transdermal fentanyl	18	13	19	24	26	37	26

Total DDD/1000HS	69	56	62	71	77	110	94

The indicators for efficiency and improvements are shown on Table 4. At the beginning of the period, the consultation team prescribed opioids to 64% of the estimated patients in need. At the end of the three year period, efficiency had risen to 87%.

**Table 4:** Proposed Indicator of efficiency and ratio of improvement in pain treatment in oncology inpatients

Indicators	2001	2002	2003	2004	2005	2006	2007
Efficiency index	37%	45%	50%	64%	61%	82%	87%
Rate of improvement	-	12%	11%	27%	-7%	54%	27%

(we estimate that 64% of oncology inpatients have to be under opioid treatment: see in the text). Efficiency index [DDD/1000HS × 640^-1 ^× 100]: Proportion of patients receiving opioid treatments in relation to the total number estimated who should be receiving them. Rate of improvement [(Final DDD/1000HS - Initial DDD/1000HS) × (640 - Initial DDD/1000HS)^-1 ^× 100]: Proportion of patients requiring opioids who began to use them in the time considered.

The rate of improvement measured as the percentage of patients who began to receive opioids of those estimated in need every year was 12%, 11% and 27% during the three years prior to the consultation team. After the consultation team started, the first year resulted in a negative growth (-7%) although it rose sharply to 54% and 27% the following two years. Overall, the rate of improvement for the three years prior and the three years following the start of the PCCT team was 43% and 64% respectively.

**Figure 1:**
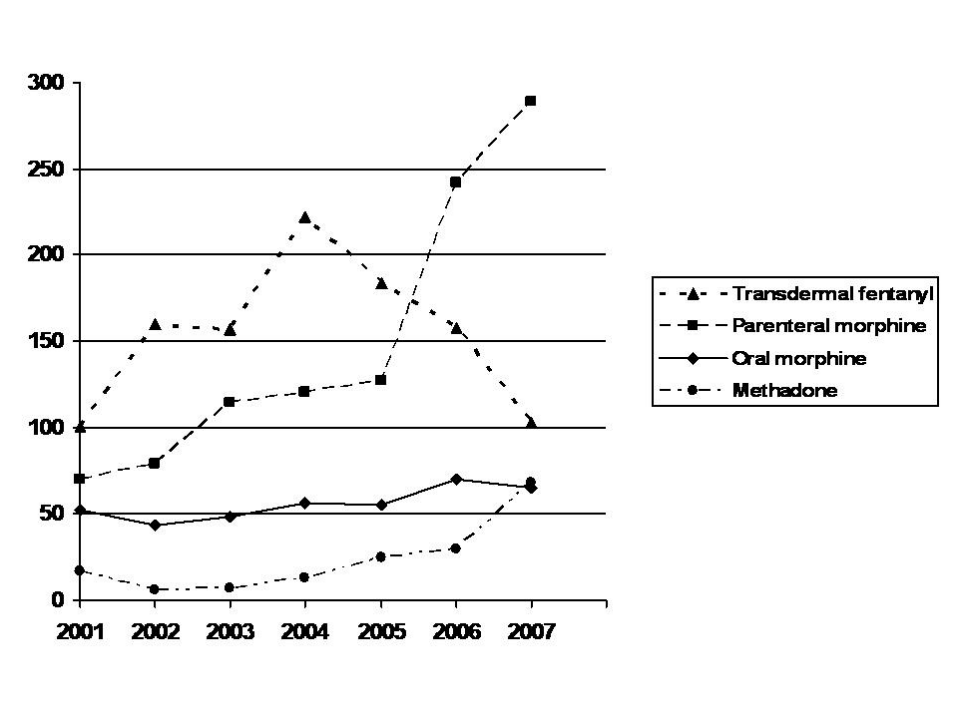
Trends in prescription of the most relevant opioids in hospitalized patients in the Oncology Department, in Daily Defines Doses per 1000 hospital stays (DDD/1000HS) per year. Palliative Care Unit start at 2004.

In regards to the trends in opioid prescription at the Oncology Department (Figure 1), the three years prior to beginning the work of the PCCT a steady increase in the prescription of both transdermal fentanyl (from 101 to 222 DDD/1000HS) and parenteral morphine (from 70 to 120 DDD/1000HS) was observed, whilst prescription of oral morphine and methadone did not change. Oxycodone had still not been released commercially.

After the beginning of the work of the consultation team in 2004, the consumption of oral methadone rose almost ten-fold (from 7 to 68 DDD/1000HS in 2007). In regards to parenteral morphine, during the PCCT's first year, prescription continued to increase steadily until it shot up the following year (from 120 to 289 DDD/1000HS). Nevertheless, a sharp fall was seen in the use of transdermal fentanyl to levels in line with those of 2001 (from 222 to 103 DDD/1000HS) whilst the prescription of oral morphine and parenteral methadone remained steady. Oxycodone was released commercially in 2004 and its consumption reached 29 DDD/1000HS in 2007. For the rest of the Hospital, the most commonly used opioids were parenteral morphine and transdermal fentanyl, with few changes over the years.

## Discussion

Expressing annual hospital opioid prescriptions in terms of the number of DDD per thousand hospital stays is a new way of presenting such data. Previous publications have used other indicators of morphine equivalent doses and generally do not standardise a particular number of individuals [26]. The concept of DDD provides an indicator of the proportion of patients receiving adequate pain treatment and how each type of opioid is used. DDD, a measurement designed for Cross-National Drug Utilization Studies [20], allows for the comparison of results internationally and between centres. In addition, the rate of efficiency described in this study offers an initial estimation of the quality of pain treatment among a group of patients. At the outset of the work of the PCCT, two out of every three patients needing opioids were receiving them. After three years, the percentage had risen to 90%. In regards to the improvement rate, in the three years prior to setting up the consultation team 43% of patients estimated in pain were initiated in opioid treatment, whilst in the three following years this figure rose to 64%. This seems to indicate that the speed at which opioid prescription increased was greater after the PCCT began its work.

It is striking that the start of the PCCT's activity did not result in an increase in the percentage of patients receiving opioids. Nevertheless, that was the same year which saw the most important change in the type of opioids prescribed: the increasing trend in the use of fentanyl declined but there was no coincident growth in the use of prescribed morphine. This suggests the existence of a sub-group of patients who are being over-treated with transdermal fentanyl. This first effect was soon compensated and the rate of growth for the following year was clearly positive, as if trying to make up lost ground. Notwithstanding, any assessment would require knowledge of the natural behaviour or development of this indicator free from the influence of a consultation team. Neither should it be assumed that the observed changes in opioid prescription are due only to the efficiency of the work of the consultation team. Since they are Oncology inpatients, it is logical to think that this is an indicator of an improvement in the knowledge and skills on pain management by the oncologists, maintaining the previous trend and also receiving the support of a new PCCT. It is easier to observe the influence of the new team in the changes in the type of opioid used and in the administration routes. The other departments in the hospital, used as control groups in the study, presented a steadily low opioid consumption. This led us to think that the changes in Oncology were not due to institutional bias, nor to changes in the policy of opioid consumption in the hospital as a whole.

A change was noted in the trend in the use of opioid formulations for Oncology inpatients. Parenteral morphine registered higher utilization than transdermal fentanyl, more than any other opioid and more than oral morphine. Morphine is considered as more appropriate than fentanyl for cancer patients in acute care centres as it allows for quicker adjustments and better management in the prevention of opioid toxicity. The higher utilization of the parenteral route rather than the oral route for morphine must to be revisited under the light of the pharmacokinetic data available and concerns over patient comfort in spite of the fact that the subcutaneous route offers an opportunity of closer contact with a registered nurse when pain control is a priority.

Higher utilization of methadone as a second-choice opioid was observed in situations which usually require consultation with the palliative care team, such as patients with neuropathic pain and cases in which opioid rotation was indicated [27]. Although methadone registered the highest percentage increase, the total amount of methadone accounted for less than 10% of total opioids prescribed. In a recent study which evaluated the prescription of long acting opioids between 1996 and 2004 in an Oncology Centre it was observed that the introduction of a Palliative Care Service coincided with a reduction in the use of fentanyl and an increase in the use of methadone [28]. The data referring to oxycodone are too premature and does not allow us to draw any conclusions; although an increase in its consumption in coming years is expected due to its role in opioid rotation in patients receiving morphine [29].

In general, it may be assumed that the profile of opioid use from 2004 onwards in this University Hospital reflects an improvement in the knowledge and skills on the use of opioids to meet the needs of cancer patients, since this is where the faster action drug and presentations are required. This change may be due not only to the direct effect of the palliative care consultation team (which cares for only 20% of the patients), but also to the influence it may have had in the Oncology Department, resulting in the prescription of the most appropriate drug profile to suit the needs of the patients.

The data from this study suggests that the implementation of PCCT in University Hospitals helps optimise the use of opioids in patients with cancer pain. Further prospective and comparative surveys on opioids prescription need to be developed in hospital settings with and without palliative care consultation teams in order to establish indicators and outcomes of efficiency of these specialized resources.

This study is aim only to evaluate the effect of a PCCT on opioid prescription. However, a PCCT should influence advanced cancer management in other profiles. Although pain is one and of the more common and relevant symptoms in Palliative Care, the origin of patient's sufferance transcends pain and the influence of other physical, social, psycho-emotional and spiritual factors that requires adequate management can be elicited. The real effect of a PCCT should be addressed including also its impact in these other areas.

## Conclusion

According to our data, the implementation of a PCCT in a University Hospital is associated with an increase in total opioid prescription in cancer patients. This increase is bound to a change in the pattern of prescription, resulting in a higher use of parenteral morphine and methadone and a decrease in transdermal fentanyl. As a whole, these changes can be interpreted as evidence not only of a higher but a more adequate use of opioids that allows a quicker adjustment and a better prevention and management of opioid toxicity.

## Competing interests

The authors declare that they have no competing interests.

## Authors' contributions

CC participated in the design and coordination of the study, and in the data analysis and interpretation. MAP participated in the collection and assembly of data and in the data analysis and interpretation. AN participated in the collection and assembly of data. AI participated in the collection and assembly of data, and interpretation. ASR: Participated in the design of the study, and in the data analysis and interpretation. All authors read and approved the final manuscript.

## Pre-publication history

The pre-publication history for this paper can be accessed here:


